# Metallic Enamel

**Published:** 1874-01

**Authors:** 


*Metallic Enamel.*—Dr. Blake, of San Francisco, professes to have discovered a method by which the crowns of teeth lost by abrasion, fracture, &c., can be restored by what is termed a metallic enamel, which is so shaded as to blend with the natural color of the tooth; it is described as follows:

Of late years the desire among dentists has been to save teeth by filling, not only when decay has taken place, but also in the case of persons of mature age, when the enamel or grinding surfaces are entirely worn off. In such instances the dentine or bony structure soon shows wear, and in a comparative short time there is not much of a tooth left above the gums. This is a source of annoyance to persons in such condition. A few years ago, when a patient's teeth were worn down or broken off, recourse was had to a metallic or rubber plate, after the bad teeth had been extracted. These plates are always more or less uncomfortable and quite expensive. Now, the art of dentistry is so perfected that the teeth can be built up, or capped with gold, giving to the patient many more years' use of his natural teeth than by the old method of substitution, or employing artificial teeth.

In front fillings, where the tooth is thus built up to its natural shape, the gold must necessarily be conspicuous, and many persons naturally object to this prominent exhibition of shining gold. This objectian, however, is now overcome entirely by the invention of Dr. Chas. E. Blake, which consists of a metallic enamel, so shading the natural color of the gold, that the bright yellow luster of the metal is changed or toned down, so as to blend with the color of the tooth. This process can be performed after the tooth has already been filled in the ordinary manner with gold. The coloring material can be inserted with the gold, and when finished, the blending together of the two metals skilfully will give any desired shade to the filling, to correspond with the color of any particular tooth. Of its durability there can be no doubt, as the material used is platinum, a white and pure metal.

No great amount of skill is required to produce a desirable shade by the alternate use of the two metals, one white and the other yellow. Gold foil is not used. Very thin plates of gold and platinum, alternating, are used and thoroughly incorporated together in filling. The platinum is plated with a thin covering of gold, so as to make it adhere closely. It is prepared in sheets from the pure metal. The finished surface is perfectly hard and solid, giving a grinding face to the tooth, which is very durable.

